# Tracheal glomus tumor misdiagnosed as pulmonary disease: a case report and literature review

**DOI:** 10.1016/j.bjorl.2021.05.011

**Published:** 2021-06-08

**Authors:** Miao Gao, Sheng-Nan Ye, Chang Lin, Yuan-Teng Xu

**Affiliations:** Fujian Otorhinolaryngology Institute, The First Affiliated Hospital of Fujian Medical University, Department of Otorhinolaryngology Head and Neck Surgery, Fujian, PR China

## Introduction

Glomus tumors (GTs) are benign tumors formed at the anastomosis of arteries and veins, affecting blood flow and temperature control. The tumor can occur in any part of the body. Owing to little data concerning tracheal glomus tumors (TGTs) of uncertain malignant potential, more accumulated cases are required to clarify its characteristics. In this report, we also review relevant literature and discuss the common features and treatments of GTs.

## Case report

**Figure 1:**
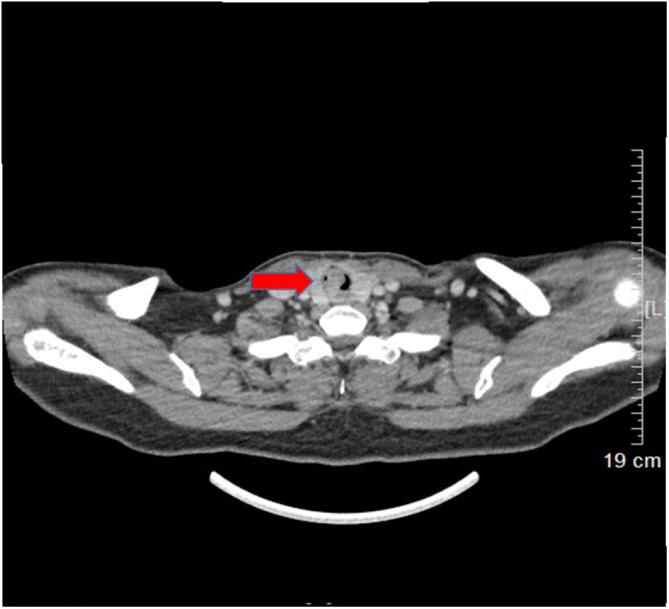
Trachea spiral CT shown a tracheal mass resulting in a 90% obstruction of trachea lumen.

**Figure 2:**
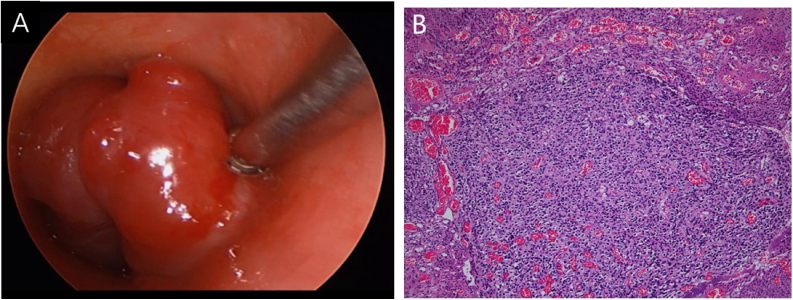
(A) The mass seen during the operation. (B) The histopathology showed well-circumscribed and solid lesion, slightly eosinophilic cytoplasm surrounded by dilated capillary-sized vessels, the cells were without obvious mitotic phase.

A 53-year-old female presented with a one-year history of cough and intermittent shortness of breath without hemoptysis or smoking history. She was admitted to the Respiratory Department due to an initial diagnosis of chronic obstructive pulmonary disease (COPD). No improvement in shortness of breath was observed after receiving long-term treatment for spasm and asthma. Spiral computed tomography (CT) with three-dimensional reconstructions in conjunction with tracheal CT enhancement scan showed enhanced tissue in the initial segment of the trachea, measuring approximately 1.4 × 0.9 × 1.4 cm (Fig. 1). A polypoid mass under the glottis almost completely blocked the lumen and moved in tandem with the patient’s breathing. The patient was referred to Otolaryngology-Head and Neck Surgery Department subsequently. To ensure the airway remained unobstructed, we performed a temporary tracheotomy. During the operation, the tumor of the subglottic area, arising from the posterior wall of the trachea was detected (Fig. 2A). The pedicle was resected, and the residual was completely cauterized with the help of Harmonic scalpel.

**Figure 3:**
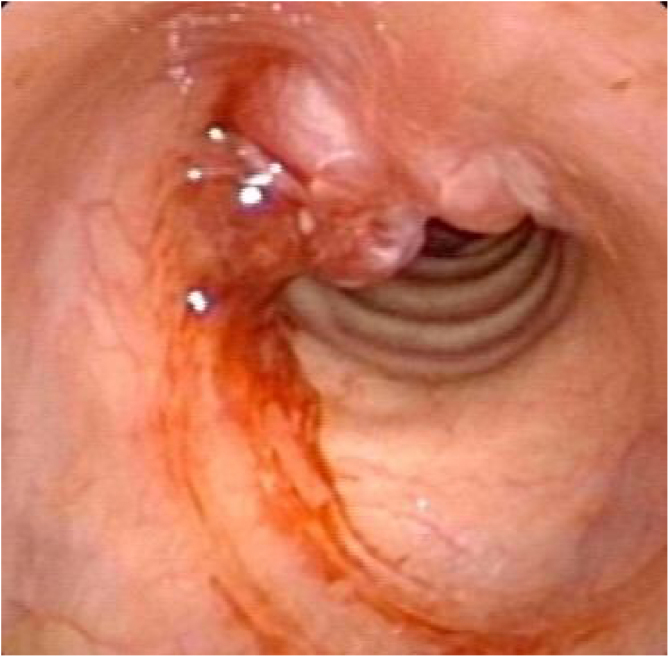
The tumor recurred in situ one year later.

**Figure 4:**
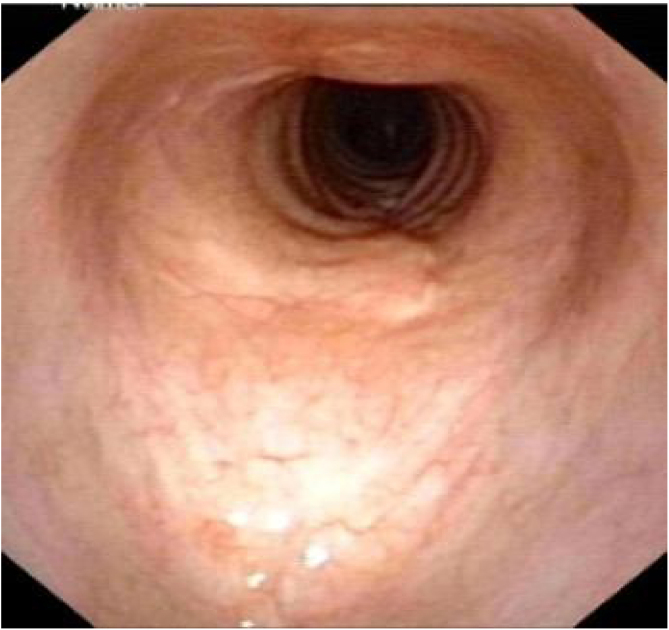
Appearance of trachea 3 month after second surgery on electronic laryngoscope.

Postoperative pathology (Fig. 2B) revealed focal eosinophilic round cells arranged like hemangiopericytoma around vessels. These cells were positive for vimentin and smooth muscle actin antibody (SMA), synaptophysin (Syn), CD56, S-100, and the Ki67 proliferation index was around 5%. Cells were negative for chromogranin-A (Cg-A), casein kinase (CK), CD34 and epithelial membrane antigen (EMA). Two weeks after the operation, a tracheal CT scan and electronic laryngoscopy were performed, demonstrating healing and scarring. The patient recovered well, and the tracheostomy tube was removed. After one year, hemoptysis occurred, and the tumor was found to have recurred (Fig. 3), expanded upward along the midline of the original tracheotomy position. The tumor and the partial tracheal sleeve resection were subsequently carried out. The patient recovered quickly after the resection and the patient is continually being monitored (Fig. 4). This study was performed under the approval of the ethics committee of our hospital (nº IEC-FOM-013-2.0).

## Discussion

Glomus tumors are rare soft tissue neoplasms derived from modified smooth muscle cells.^1^ GTs were first described by Masson in 1924. The first case was reported in 1950.^2^ Based on the World Health Organization (WHO) classification of tumors, GTs are commonly classified into three groups including benign GTs, GTs of uncertain malignant potential, and malignant GTs.^3^ According to the recent WHO classification, the criteria for tumors of uncertain malignant potential is contingent on not fulfilling the criteria for malignancy, and additionally exhibiting at least one atypical feature other than nuclear pleomorphism.^4^ However, GTs > 2 cm in size with a deep location which were previously diagnosed as malignant are now classified as having uncertain malignant potential.^3^ In the present case, the tumor was large enough but did not show significant mitotic characteristics or nuclear atypia, thus the diagnosis of uncertain malignant potential.

Clinically, symptoms associated with airway irritation are common in bronchial and tracheal GTs, but asymptomatic GT usually occurs in the peripheral pulmonary parenchyma.^5^ Depending on the pathology, the main differential diagnoses of tracheal glomus tumor are carcinoid tumor and hemangiopericytoma. CT scan and bronchoscopy are the best diagnostic methods for determining the origin of the tumor currently. However, the complete diagnosis depends on the results of a pathological examination. Due to their abundant vessels, tracheal glomus tumors (TGTs) display an obvious enhanced area in CT images.^6^ Despite this, it is difficult to distinguish GTs from carcinoid or hemangiomas solely based on radiologic findings as they appear to have a well-circumscribed round mass under contrast enhancement.^7^ Thus, the identification of the cytological and vascular structural characteristics are particularly important for an accurate classification.

While there is currently no consensus on the treatment of TGTs. Masoum et al.^8^ reported a case of a 21-year-old patient who underwent GT resection via bronchoscopy, which recurred one year later, followed by subsequent open resection. Jin et al.^5^ preferred open surgery due to the young age of the patient and the sizable nature of the lesion. In that case, the risk of bleeding and high recurrence was reduced. Recently, Suresh et al.^9^ introduced a novel treatment for thoracic tracheal GTs, named percutaneous trans-tracheal endoscopic approach (PTEA). The procedure has many obvious advantages in the resection of benign lumen lesions of the lower trachea, it is an easy and better controlled, simple, and less morbid procedure, but further studies are needed to determine the practicability and safety of the method. Hartert et al.^10^ described a patient who was treated by tracheal sleeve resection via a right posterolateral thoracotomy with end-to-end anastomosis, citing a 96 months followup period without recurrence. Sleeve resection with primary reconstruction of the trachea is the treatment of choice for tracheal glomus tumor.^1^ So in the second operation, we adopted this approach. The patient recovered well.

To the best of our knowledge, there are 77 published articles regarding GT in the English language medical literature (Table 1), most of the relevant articles being case reports. The most common symptoms reported among symptomatic GT patients are cough 54.55% (42/77), dyspnea 54.55% (42/77) and hemoptysis 44.16% (34/77). Patients with a lower frequency of chest pain (7.9%) were more rarely reported and presented chest tightness, fever, and asthma-like symptoms. Only five patients were reported to be asymptomatic. The locations of TGTs were of superior origin in 20.78% (16/77) of cases, middle in 23.38% of cases (18/77), inferior in 35.06% of cases (27/77), and bronchial in 20.78% of cases (16/77) (Table 2). These distributions suggest that incidences of GT in the lower two-thirds of the trachea are commonplace, perhaps due to the numerous mucous glands and vessels. Most of the published GT cases are benign, with only two cases of uncertain malignant potential previously reported.^5^ The tumors in these cases were located at the lower third of the trachea or bronchus, both removed by open surgery with no recurrence after two-year follow-up. Except for the location of the bronchial section, a majority of tumors were surgically removed. Only four bronchial GT patients underwent open surgery. Meanwhile, two patients underwent partial or total lung resection. A few patients accepted adjuvant treatments after surgery, including radiochemotherapy, cryotherapy, Nd-YAG laser and argon plasma coagulation (Table 2).

**Table 1:** Summary of literature of tracheal glomus tumor.

First author	Year	Age (years)	Sex	Symptoms	Duration of symptoms before treatment	Tumor site (S/M/I/B)	Size (cm)	Treatment	Follow-up
Hussarek	1950	43	F	Dyspnea	Not stated	S	Bean-sized	Tracheal resection	Not stated
Fabich	1980	63	M	Cough	2 years	I	2.5 × 2.0 × 1.0	Sleeve resection	Died of complications on the 10^th^ post-op day
Warter	1980	69	M	Dyspnea, hemoptysis	Not stated	M	2.3 × 1.5 × 1.5	Segmental resection	Unremarkable
Heard	1982	50	M	Dyspnea	Not stated	I	2.5 × 1.5 × 1.0	Sleeve resection	Sepsis, died on the 15^th^ post-op day
Ito	1988	51	M	Hemoptysis	9 months	S	1.5 × 1.2 × 1.0	Segmental resection	2 years
Sheffield	1988	74	M	Cough, dyspnea	<1 month	I	2.2	Endoscopic resection	7 months
Kim	1989	54	F	Cough, dyspnea, hemoptysis	3 years (cough)	M	1.5 × 1.2	Segmental resection	13 months
Shin	1990	47	F	Cough, hemoptysis	3 years	I	1.5 × 1.0 × 1.0	Wedge resection	Not stated
GarciaPrats	1991	58	M	Cough, dyspnea, hemoptysis	Several years	M	2.5 × 1.8	Segmental resection	8 months
Haraguchi	1991	61	M	Asymptomatic	Asymptomatic	M	1.2	Sleeve resection	Not stated
Arapantoni	1995	65	M	Dyspnea, hemoptysis	3 months (dyspnea), 3 days (hemoptysis)	I	4.5 × 3.0	Endoscopic resection and Nd-YAG	1 year
Koskinen	1998	66	M	Asymptomatic	Not stated	I	2.0 × 3.0	Endoscopic resection, Nd-YAG and external radiotherapy	Not stated
Watanabe	1998	43	M	Hoarseness	Not stated	I	2.0 × 1.6 × 1.4	Sleeve resection	20 months
First author	Year	Age (years)	Sex	Symptoms	Duration of symptoms before treatment	Tumor site (S/M/I/B)	Size (cm)	Treatment	Follow-up
Menaissy	2000	34	M	Hemoptysis	2 months	M	2.4 × 2.1 × 1.6	Tracheal resection	4 months
Lange	2000	20	M	Asthma-like symptoms	< 1 month	B	1.4 × 1.3 × 0.6	Bronchial sleeve resection	9 months
Oizumi	2000	48	M	Hemoptysis	Not stated	B	0.7	Bronchial resection	3 months
Gowan	2001	73	M	Chest pain, dyspnea, hemoptysis	5 weeks	M	1.6 × 0.3 × 0.6	Segmental resection	6 years
Chien	2003	50	F	Cough, dyspnea, hemoptysis	8 years (cough and dyspnea), 1 day (hemoptysis)	I	2.5 × 2.5 × 2.0	Segmental resection	1 year
Vailati	2004	40	M	Dyspnea, cough, fever	6 months	B	5.0 × 1.5	Endoscopic resection	1 month
De Weerdt	2004	37	M	Dyspnea, cough, fever	2 months	B	Not stated	Endoscopic resection + cryotherapy + Nd-YAG laser	3 months
Nadrous	2004	39	M	Hemoptysis	30 months	S	2.0 × 1.5 × 1.5	Sleeve resection	3 months
Ren	2005	29	M	Cough, dyspnea	2 years (cough), 2 months (dyspnea)	I	1.7 × 2.0 × 1.7	Segmental resection	Not stated
Takahashi	2005	67	M	Cough	Not stated	B	0.8	Bronchial resection	Not stated
Altinok	2006	83	F	Dyspnea, hemoptysis	3 months	S	2.0 × 1.5 × 1.2	Partial sleeve resection	1 year
Haver	2008	10	F	Dyspnea, chest pain, cough	3 weeks	M to I	1.8 × 1.3 × 1.3	Tracheal resection	2 years
Colaut	2008	70	M	Dyspnea, wheezing	2 months	M	2.0 × 1.0 × 1.0	Endoscopic resection and Nd-YAG	2 years
Akata	2008	39	M	Cough	<1 month	B	2.5 × 2.5 × 2.0	Endoscopic resection	6 years
First author	Year	Age (years)	Sex	Symptoms	Duration of symptoms before treatment	Tumor site (S/M/I/B)	Size (cm)	Treatment	Follow-up
Shang	2010	59	M	Chest pain, dyspnea cough	10 years	I	2.0 × 1.0 × 0.5	Endoscopic resection + electrocautery	1 year
Shang	2010	22	F	Cough, hemoptysis dyspnea	1 year	I	1.8 × 1.5 × 1.4	Endoscopic resection + electrocautery	1 year
Nakajima	2010	30	M	Hemoptysis	6 months	B	1.5 × 1.3	Bronchial resection	10 months
Parker	2010	43	F	Dyspnea, chest pain, cough	6 months	I	2.0 × 1.6 × 1.5	Tracheal resection	11 months
Baek	2011	54	M	Dyspnea, cough	3 months	M	1.3 × 1.2	Tracheal resection	2 years
Mogi	2011	56	F	Cough, dyspnea	7 months	I	1.3 × 1.2 × 1.1	Tracheal sleeve resection	9 months
Ravenna	2011	79	F	Dyspnea, cough	3 months	B	Not stated	Endoscopic resection + Nd-YAG laser	5 years
Sakr	2011	66	M	Cough, dyspnea	2 months (cough), 10 days (dyspnea)	S	1.2 × 0.8 × 2.0	Endoscopic resection + tracheal sleeve resection	21 months
Okereke	2011	58	M	Dyspnea	Long term	M	1.1	Tracheal resection	6 months
Norder	2012	49	F	Cough, dyspnea	3 years	S	1.2 × 1.1 × 1.1	Endoscopic resection	Not stated
Lange Lazdunki	2012	62	F	Cough, dyspnea	Not stated	I	1.6	Left upper lung resection	Not stated
Cukurova	2012	50	M	Cough, dyspnea, hemoptysis	Not stated	S	Not stated	Endoscopic resection	3 years
Ariizumi	2012	43	F	Asymptomatic	3 months	B	Not stated	Tracheal resection	6 months
First author	Year	Age (years)	Sex	Symptoms	Duration of symptoms before treatment	Tumor site (S/M/I/B)	Size (cm)	Treatment	Follow-up
Zhu	2013	30	F	Dyspnea, hemoptysis	1 year	B	4.0 × 0.5 × 0.5	Tracheal resection	18 days
Fan	2013	15	M	Cough, dyspnea, hemoptysis	3 months	M	2.0 × 2.5	Tracheal resection	1 year
Ghigna	2013	70	M	Hemoptysis	Not stated	I	1.6	Tracheal resection	Not stated
Ghigna	2013	40	M	Hemoptysis	Not stated	I	1.0	Tracheal resection	Not stated
Chang	2013	76	M	Fever	1 week	M	Not stated	Endoscopic resection	Not stated
Singh	2013	65	F	Cough	3 months	B	1.2 × 0.4 × 0.5	Endoscopic resection	Not stated
Wei	2013	39	M	Cough, hemoptysis	1 year	S	1.9 × 1.4 × 0.8	Tracheal resection	26 months
Wei	2013	43	M	Dyspnea	3 years	I	2.0 × 1.5	Tracheal resection	19 months
Choi	2014	52	F	Asymptomatic	Asymptomatic	B	1.6	Resection of carina and both main bronchi	3 months
Choi	2014	64	M	Asymptomatic	Asymptomatic	M	2.6	Tracheal resection	2 years
Xiong	2014	48	F	Cough, dyspnea	6 years	I	1.2 × 1.0 × 0.8	Bronchoscopic cryoablation and argon plasma coagulation (APC)	6 months
Xiong	2014	55	M	Cough, dyspnea; chest pain	13 days (Hemoptysis); 5 months (Cough and chest pain)	I	0.5 × 0.3 × 0.3	Bronchoscopic cryoablation and argon plasma coagulation (APC)	6 months
Wu	2014	58	F	Hemoptysis	Not stated	I	2.2 × 2.2	Tangential resection with spiral tracheoplasty	2 years
Zhang	2014	54	M	Cough, hemoptysis	4 years	B	2.5 × 1.5 × 1.0	Right total lung resection	6 months
First author	Year	Age (years)	Sex	Symptoms	Duration of symptoms before treatment	Tumor site (S/M/I/B)	Size (cm)	Treatment	Follow-up
Zhang	2014	48	M	Cough	1 year	B	Not stated	Right upper lung lesion resection	7 months
Huang	2015	39	F	Dyspnea	More than 1 year	S	2.5 × 1.2	Segmental resection	1 month
Liu	2015	39	F	Dyspnea	More than 1 year	S	2.5 × 1.2	Segmental resection	1 month
Li	2015	15	M	Cough, hemoptysis	2 months	M	1.2 × 1.0 × 1.0	High-frequency electrocautery and APC	3 months
Tan	2015	44	M	Cough, dyspnea, hemoptysis	2 months	I	3.0 × 2.5 × 1.0	Tracheal resection	20 months
Masoum	2015	21	M	Cough, hemoptysis	Several months	S	Not stated	Endoscopic resection + tracheal resection	1 year
Fernandez-Bu	2015	48	M	Hemoptysis and cough	3 months	I	2.0 × 2.0	Endoscopic resection	2 years
Brzezinski	2015	38	M	Dyspnea	1 year	S	1.6 × 1.8 × 0.8	Tracheal resection	Not stated
Rashid	2015	52	M	Hemoptysis	3 months	B	Not stated	Endoscopic resection	6 months
Xiong	2016	52	F	Dyspnea, cough	6 months	S	2.0 × 1.0 × 1.0	High-frequency electrocautery and APC	9 months
Aryan	2016	50	F	Hemoptysis, cough, dyspnea	1 week	B	Not stated	Endoscopic resection	Not stated
Wang	2016	63	M	Hemoptysis	1 week	I	0.5 × 0.3	High-frequency electrocautery and APC	15 months
Wang	2016	44	M	Hemoptysis, cough	1 week	M	1.0 × 1.5	Endoscopic resection + tracheal resection	8 months
Venegas	2017	51	F	Dysphagia, hemoptysis dyspnea	Several weeks	I	2.6 × 2.3 × 1.7	Tracheal resection;	12 months
First author	Year	Age (years)	Sex	Symptoms	Duration of symptoms before treatment	Tumor site (S/M/I/B)	Size (cm)	Treatment	Follow-up
Huang	2017	38	M	Cough, hematemesis	20 days	M	2.4 × 2.2 × 2.7	The video-assisted transthoracic surgery (VATS)	3 months
Suresh	2018	43	M	Dyspnea	Not stated	M	Not stated	Percutaneous trans-tracheal endoscopic approach (PTEA)	Not stated
Gou	2018	30	M	Cough, expectoration	1 month	S	2.0	High frequency electroexcision	1 year
Gou	2018	47	M	Hemoptysis	3 years	I	1.8	Tracheal resection, and anastomosis	1 year
Jin	2019	51	M	Cough	4 months	I	2.0	Segmental tracheal resection	2 years
Hartert	2019	66	M	Cough, hemoptysis dyspnea	3 months	I	1.1 × 2.2	Thoracotomy with end-to-end anastomosis	96 months
Shao	2020	41	M	Chest tightness; chest pain; hemoptysis	1 month (chest tightness; chest pain); hemoptysis (2 weeks)	s	1.0 × 1.2 × 1.2	Endoscopic resection	Not stated
Present case	2020	53	M	Cough, dyspnea	1 year	S	1.4 × 1.4 × 0.9	Harmonic scalpel with Video endoscope	11 months

**Table 2:** Different parts of the treatment.

Location	Number	%	Endoscopic	Surgery	High frequency eletrocautery	Others (like APC Nd-YAG laser)
S	16	20.78	6	8	2	1
M	18	23.38	3	14	1	2
I	27	35.06	6	18	3	5
B	16	20.78	11	4	0	2

S, superior; M, medium; I, inferior; B, bronchi.

We suggest that as respiratory symptoms cannot be effectively resolved, it may be necessary to perfect examinations for rare diseases conscientiously, especially GTs can be easily missed and misdiagnosed. Although there is no cure in this case, initial bronchoscopy intervention plays a key role in timely and effectively restoring the airway of patients with severe symptoms and providing preoperative diagnostic information. The bronchoscopy biopsy should be avoided due to the tumor’s rich vasculature. Complete resection of the tumor remains the basic procedure of treatment. Long-term followup of tracheal conditions after surgery is certainly necessary.

## Conclusion

Tracheal GTs of uncertain malignant potential, while uncommon, currently have no uniform standards for the surgical treatment of GTs and can be easily mistaken for a pulmonary disease if the symptoms are atypical, we should pay attention to it. Radical resection is still worthy of consideration because of the possibility of recurrence in the clinic. Additionally, tracheal stenosis is the most likely complication of concern.

## Conflicts of interest

The authors declare no conflicts of interest.
